# Imaging Live Cells at the Nanometer-Scale with Single-Molecule Microscopy: Obstacles and Achievements in Experiment Optimization for Microbiology

**DOI:** 10.3390/molecules190812116

**Published:** 2014-08-13

**Authors:** Beth L. Haas, Jyl S. Matson, Victor J. DiRita, Julie S. Biteen

**Affiliations:** 1 Department of Chemistry, University of Michigan, Ann Arbor, MI 48019, USA; E-Mail: leveret@umich.edu; 2 Department of Medical Microbiology and Immunology, University of Toledo, Toledo, OH 43606, USA; E-Mail: Jyl.Matson@utoledo.edu; 3 Department of Microbiology and Immunology, University of Michigan, Ann Arbor, MI 48019, USA; E-Mail: vdirita@umich.edu

**Keywords:** single-molecule microscopy, super-resolution imaging, single-particle tracking, fluorescence, microbiology, live-cell imaging

## Abstract

Single-molecule fluorescence microscopy enables biological investigations inside living cells to achieve millisecond- and nanometer-scale resolution. Although single-molecule-based methods are becoming increasingly accessible to non-experts, optimizing new single-molecule experiments can be challenging, in particular when super-resolution imaging and tracking are applied to live cells. In this review, we summarize common obstacles to live-cell single-molecule microscopy and describe the methods we have developed and applied to overcome these challenges in live bacteria. We examine the choice of fluorophore and labeling scheme, approaches to achieving single-molecule levels of fluorescence, considerations for maintaining cell viability, and strategies for detecting single-molecule signals in the presence of noise and sample drift. We also discuss methods for analyzing single-molecule trajectories and the challenges presented by the finite size of a bacterial cell and the curvature of the bacterial membrane.

## 1. Introduction

Single-molecule fluorescence microscopy enables the investigation of biological questions in living cells at millisecond- and nanometer-scale resolution. Myriad existing reviews of single-molecule microscopy (see for example references [1,2,3,4]) cover a variety of methods and applications for specialists in the field. However, as single-molecule super-resolution imaging and tracking become more accessible to non-experts, the experimental considerations are of great importance to a larger number of scientists. In particular, though a single-molecule approach is a relatively straightforward way to implement super-resolution imaging using a conventional microscope, optimizing new single-molecule experiments can be challenging. In this review, we summarize common obstacles to live-cell single-molecule microscopy and describe methods we have developed and used to overcome these challenges in live bacteria. We examine the choice of fluorescent molecule (fluorophore) and labeling scheme, approaches to achieving single-molecule levels of fluorescence, considerations for maintaining cell viability, and strategies for detecting single-molecule signals in the presence of noise and sample drift. We also discuss methods for analyzing single-molecule movement and the specific challenges encountered when investigating bacteria cells, which are small in size and have highly curved membranes. These considerations are discussed in the context of our recent work on discerning the mechanism of membrane-bound transcription regulation in the control of virulence in the human pathogen *Vibrio cholerae*, as well as in terms of studies of other systems from the literature, in order to illustrate the obstacles encountered when designing single-molecule microscopy experiments in live bacteria. This review is directed to students, non-specialists, and others who may have more interest than expertise in designing a single-molecule microscopy experiment.

### 1.1. Conventional Limits: The Diffraction Limit of Light

**Figure 1:**
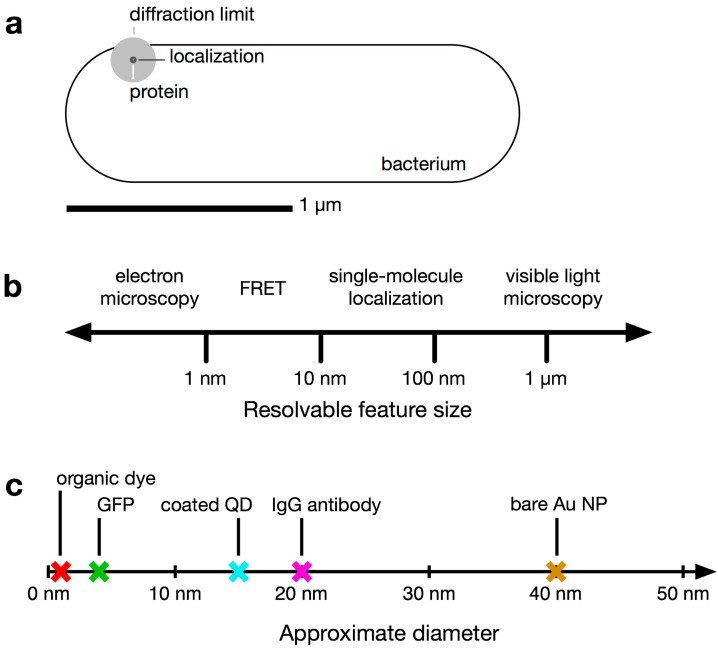
Relevant size scales in subcellular imaging of bacteria. (**a**) Comparison of the size of a *V. cholerae* bacterial cell and the diffraction limit of light. A small fluorescent protein appears blurred to the diffraction limit (~200 nm, gray circle). Single-molecule imaging localizes the fluorophore with greater precision (~30 nm, black circle); (**b**) Resolvable size scales (log scale) using several common microscopy techniques; (**c**) Approximate diameters of an organic fluorophore, a fluorescent protein (GFP [8]), a coated quantum dot (QD; [9]), an immunoglobulin (IgG) antibody [10], and a 40-nm gold nanoparticle (NP).

Light microscopy is non-invasive, minimally perturbative, and non-toxic to live samples at low irradiation intensities. However, because the diffraction of light limits the resolution of a microscope [5], no matter how powerful the magnification of the objective lens, even an infinitesimally small point source will produce an image of finite size, called the point-spread function (PSF) of the microscope. Two points placed very close together will have overlapping PSFs. The minimum separation distance at which the points can be resolved is the Abbe limit, which depends on the wavelength of the emitted light (390–700 nm for light in the visible spectrum) and the numerical aperture (NA) of the microscope objective [5]. For example, the smallest yellow (λ = 560–580 nm) feature resolvable through an objective with a large NA (e.g., 1.4) is more than 200 nm in size. Objects closer than this limit are blurred together by the diffraction limit of light and are therefore unresolvable. Even large proteins are much smaller than this 200-nm limit, precluding direct observations of protein conformations and interactions by conventional optical microscopy (Figure 1a). Greater precision can be achieved by using smaller wavelengths; for example, an electron microscope can reach sub-nanometer resolution (Figure 1b [6]), but such methods are not compatible with live biological samples, so dynamic information is lost. Other methods, such as Förster Resonance Energy Transfer (FRET, Figure 1b [7]), can report on the nanoscale proximity of two fluorescent molecules, but single-molecule FRET has found limited applications in live bacteria to date.

### 1.2. Super-Resolution Microscopy

Super-resolution techniques have been developed to overcome the diffraction limit of light. In single-molecule fluorescence imaging, target molecules are labeled with fluorescent tags and observed one at a time in space or time, and the observed fluorescence intensity profiles are fit with a model function to localize each molecule with much greater precision than traditional light microscopy can achieve [11]. Super-resolution imaging is achieved when the positions of a distribution of molecules are built up over time. Common variations of this technique include Photoactivated Localization Microscopy (PALM; [12]), Fluorescence Photoactivation Localization Microscopy (FPALM; [13]), and Stochastic Optical Reconstruction Microscopy (STORM; [14]). The image acquisition time here is generally on the scale of minutes, though recent developments have enabled second-scale super-resolution imaging [15]. Furthermore, single-molecule tracking permits super-resolution information about molecular motions to be attained. This method was introduced as Fluorescence Imaging with One-Nanometer Accuracy (FIONA; [16]), though many variations on super-resolution tracking techniques exist.

In addition to providing high-precision position information, single-molecule imaging and tracking have been combined with three-dimensional imaging and multicolor or multi-emitter detection (Table 1). Combining multiple imaging modalities enables super-resolution microscopy to access increasingly complex systems. Regardless of the configuration, each of these single-molecule microscopy experiments must be optimized for the specific goals of the investigation; this includes a careful choice of the fluorophore, the labeling scheme, and the imaging parameters. Other super-resolution microscopy methods (Table 1), such as Stimulated Emission Depletion (STED; [17]) and Structured Illumination Microscopy (SIM; [18]) employ patterned excitation rather than localized single-molecule emission, and are reviewed elsewhere [2,19].

**Table 1:** **Top:** Single-molecule fluorescence-based super-resolution imaging and tracking techniques in order of publication. **Bottom:** Other optical microscopy methods that have been used in live cells and that are discussed in the text.

Method	Full Name	Reference
FIONA	Fluorescence Imaging with One-Nanometer Accuracy	[16]
SHRImP	Single-molecule High-Resolution Imaging with Photobleaching	[20]
NALMS	Nanometer-Localized Single-Molecule Fluorescence Microscopy	[21]
SHREC	Single-molecule High-Resolution Colocalization	[22]
ICA	Superresolution by Localization of Quantum Dots Using Blinking Statistics	[23]
PALM	Photoactivated Localization Microscopy	[12]
STORM	Stochastic Optical Reconstruction Microscopy	[14]
FPALM	Fluorescence Photoactivation Localization Microscopy	[13]
PAINT	Point Accumulation for Imaging in Nanoscale Topography	[24]
PALMIRA	PALM with Independently Running Acquisition	[25]
dSTORM	Direct STORM	[26]
uPAINT	Universal PAINT	[27]
CALM	Complementation Activated Localization Microscopy	[28]
BALM	Binding-Activated Localization Microscopy	[29]
SPRAIPAINT	Superresolution by Power-Dependent Active Intermittency PAINT	[30]
BaLM	Bleaching/blinking assisted Localization Microscopy	[31]
SMACM	Single-Molecule Active-Control Microscopy	[32]
TALM	Tracking and Localization Microscopy	[33]
FRET	Förster Resonance Energy Transfer	[7]
FCS	Fluorescence Correlation Spectroscopy	[34]
FRAP	Fluorescence Recovery After Photobleaching	[35]
STED	Stimulated Emission Depletion	[17]
(S)SIM	Saturated Structured-Illumination Microscopy	[18]

### 1.3. Bacteria Beyond the Diffraction Limit

Imaging molecules inside bacteria presents additional challenges for super-resolution imaging. First, bacteria are small (generally 1–10 µm long [36]). A 2-µm *Vibrio cholerae* bacterium, for example, is only 10 times as long as the diffraction limit [37], making sub-cellular resolution a challenge (Figure 1a). Second, bacteria lack most of the organelles of eukaryotes, so cellular functions, from metabolism to transcription, are carried out in the cytoplasm [36]. Soluble cytoplasmic proteins diffuse very rapidly in three dimensions (e.g., in the *Escherichia coli* cytoplasm, enhanced yellow fluorescent protein (EYFP) has a diffusion coefficient of ~7 µm^2^/s [38]), which makes these molecules difficult to track, even with single-molecule tracking. Third, the bacterial membrane surface is highly curved, such that even slow-moving membrane-associated diffusion appears distorted when captured on a two-dimensional detector [4,39].

Early bacterial microscopy studies focused on cell morphology [40,41], and staining methods, such as the Gram test, informed investigations into bacterial membrane structure [42]. Bulk fluorescence methods, such as Fluorescence Correlation Spectroscopy (FCS; [34]) and Fluorescence Recovery After Photobleaching (FRAP; [35]) provide views of the average subcellular motions, but a truly molecular-scale picture of live, dynamic cells has only emerged in the 21st century [16,43]. Even now, with single-molecule resolution imaging available, most live-cell studies of bacteria explore model systems (e.g., *E. coli*, *Caulobacter* *crescentus* [1,3,44,45]).

### 1.4. Case Study: Membrane-bound Transcription Regulation in the Human Pathogen V. cholerae

**Figure 2:**
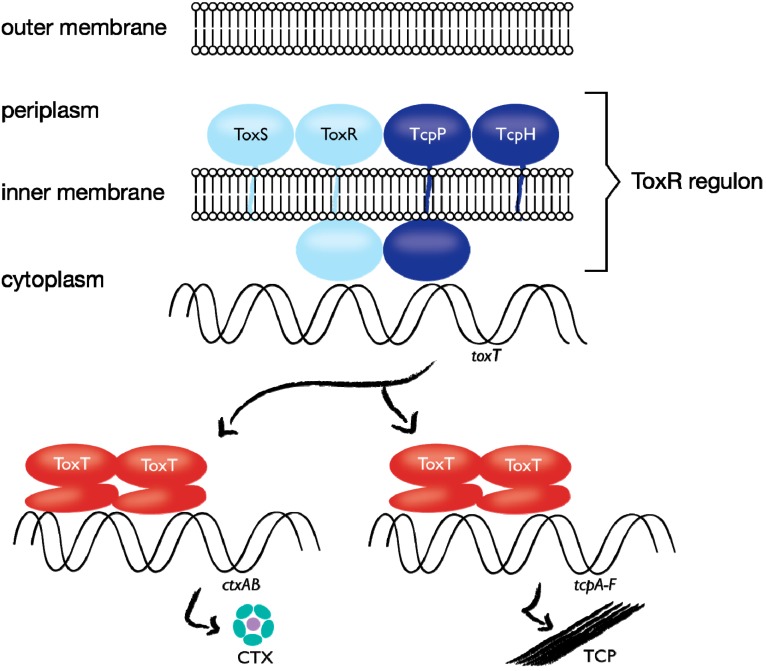
Role of the ToxR regulon in controlling cholera toxin (CTX) expression. The inner-membrane proteins TcpP and ToxR bind DNA at the *toxT* promoter, and activate transcription of *toxT*. ToxS and TcpH contribute to the function or stability of ToxR and TcpP, respectively. ToxT activates *ctxAB* and *tcpA–F* transcription. Figure modified from reference [47].

Expression of cholera toxin (CTX) by the human pathogen *V. cholerae* is controlled by an unusual set of membrane-localized transcription activators, the regulatory output of which is collectively termed the ToxR regulon (Figure 2). We have used single-molecule fluorescence imaging to investigate the diffusion of the membrane-bound transcription activator protein TcpP in live *V. cholerae* cells [46], and this work illustrates the obstacles encountered when designing single-molecule microscopy experiments in live bacteria to push beyond the traditional diffraction limit.

The direct virulence gene regulator of *V. cholerae* pathogenicity is ToxT, which activates expression of genes encoding cholera toxin (*ctxAB*) and toxin-coregulated pilus (*tcpA-F*) [47]. Activation of *toxT* gene expression is itself controlled by the action of two membrane-localized transcription activators, ToxR and TcpP. Prior to the discovery in *V. cholerae* of ToxR, a membrane protein capable of binding and activating transcription, it was widely assumed that transcription factors localize to the cytoplasm, where DNA and other components of the transcription apparatus, such as RNA polymerase, reside. Thus *V. cholerae* has been an important subject of biological research both because of its pathogenicity and because it is a model for DNA binding and gene regulation controlled by the membrane proteins ToxR and TcpP [48,49]. Indeed, membrane-localized transcription is not unique to *V. cholerae*: subsequently, several other prokaryotic organisms have been discovered to employ this mechanism (Table 2).

**Table 2:** Membrane-localized transcription activators have been identified in several different bacterial species, as enumerated below. Membrane-localized transcription has also been observed in the archaea *Sulfolobus* *acidocaldarius*.

Species	Proteins	References
*Vibrio cholerae*	ToxR	[48]
	TcpP	[49]
	CadC	[50]
	TfoS	[51]
*Vibrio fischeri*	LuxR	[52]
	ToxR	[53]
*Vibrio parahaemolyticus*	ToxR	[54]
*Escherichia coli*	CadC	[55]
*Bacteroides thetaiotaomicron*	SusR	[56]
*Yersinia pseudotuberculosis*	PsaE	[57]
*Photobacterium* spp*.*	ToxR	[58]
*Salmonella typhimurium*	MarT	[59]
*Sulfolobus acidocaldarius*	ArnR	[60]

To study these activators with single-molecule resolution, TcpP was labeled at the periplasmic C-terminus with a fluorescent protein, such that its diffusion could be tracked without disrupting DNA-binding at the cytoplasmic N-terminus [49]. Bacterial cultures of O395 Δ*tcpP* TcpP-PAmCherry *V. cholerae* [46] were grown in LB rich medium with kanamycin (50 µg/mL) at 37 °C with 180 rpm shaking. Cultures were transferred to M9 minimal medium with 0.4% glycerol and 25 m*M* amino acid supplement (asparagine, arginine, glutamic acid, and serine) and grown to turbidity at 30 °C with 180 rpm shaking. To induce TcpP-FP expression, the cultures were incubated in 0.1% arabinose for an additional 3–4 h.

## 2. Fluorescent Labels

Single-molecule imaging overcomes the challenge posed by the diffraction limit of light by precisely determining the position of each isolated fluorescent emitter. The precision with which each molecule can be localized depends on the square root of the number of emitted photons detected [11]; the more photons collected, the better the localization precision. Intracellular investigations are not limited to intrinsically fluorescent molecules: most fluorescence imaging studies require that the target of interest be identified by a fluorescent label. To ensure high-resolution images, the choice of fluorophore is thus very important. Furthermore, background signals from cellular autofluorescence, diffuse fluorescence from very fast-moving molecules, excess or unwanted fluorophores incorporated into the cell, and camera noise can all worsen the localization precision. *In vitro* single-molecule experiments achieve high signal-to-noise ratios based on imaging bright, stationary fluorophores in controlled environments, but in live-cell experiments, fluorophores may be dim, background fluorescence can be high, and the molecules of interest generally move during observation [11,61,62,63]. Fluorophore bleaching and blinking can also reduce detectability.

### 2.1. General Considerations

**Figure 3:**
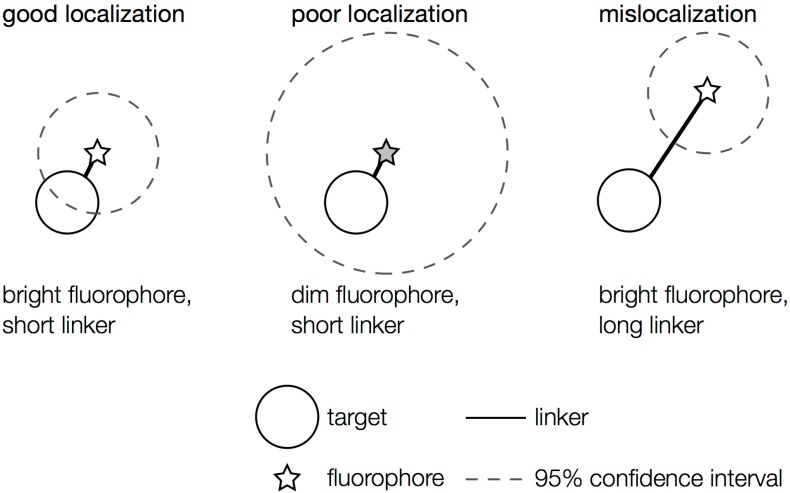
Localization problems arising from dim fluorophores or long linkers. A bright fluorophore that is near the target of interest provides the most precise localization (dashed circle: small confidence interval). Dimmer fluorophores are localized less well (dashed circle: large confidence interval). A precisely localized fluorophore that is far from the feature it is intended to label will not report the actual position accurately.

When choosing the appropriate fluorophore and labeling scheme for a particular live-cell imaging experiment, both the fluorescent properties of the tag and the effect of the label on the organism must be considered. Ideally, the fluorophore used should report the position of its target very precisely without perturbing the system in any way. If the linker between the fluorophore and its target is too long, as can be the case, for instance, for antibody labeling, which inserts a 20-nm space between the target and the label [64], mislocalizations (aberrant localizations) may occur: the fluorophore may be localized with high precision, but because the probe is several nanometers from the point of interest, the uncertainty in the target position is much greater than the fluorophore localization precision suggests (Figure 3). Additionally, because the diffusion coefficient is inversely related to particle size [65], large tags may hinder the diffusion of small, mobile molecules in cells.

In addition to local effects, it is important to determine that labeling the target molecule does not interfere with the normal functions of the protein, or with larger-scale cellular functions. Whenever possible, labeled cells should grow at rates similar to unlabeled cells and exhibit wild type phenotypes. Measurement of protein activity is crucial. For example, in our studies of *V. cholerae* expressing TcpP-PAmCherry as the sole TcpP source, we identified TcpP-PAmCherry induction conditions that led to wild-type expression levels of the toxin coregulated pilus protein TcpA, whose expression is regulated by TcpP (Figure 2). However, the PAmCherry fusion did behave slightly differently than wild type TcpP in some respects. Notably, while the TcpH protein is required to protect wild-type TcpP from degradation (Figure 2; [66]), TcpP-PAmCherry stability is less dependent on TcpH [46]. Here, the fluorescent protein tag in the periplasm may mimic the stabilizing role of TcpH. Other examples of careful consideration of biological activity after introducing labels were performed by Xie *et al.*, who found that *E. coli* T7 RNAP labeled with the yellow fluorescent protein Venus at the N-terminus maintains its polymerase activity; that the protein Tsr maintains the ability to enter to membrane when labeled at its C-terminus [1,67]; and that labeling the *E. coli lac* repressor does not impede LacI DNA-binding activity, but that the tagged repressor forms LacI dimers, rather than the LacI tetramers present in wild type [68].

Due to the requirements of small size, labeling specificity and maintaining cellular function, the most commonly used fluorophores in live-cell single-molecule localization microscopy experiments are fluorescent proteins. Small-molecule dyes attached using enzymatic labeling schemes or antibody labeling, and other labels, such as unnatural amino acids and quantum dots, have also been used [1,2,3,4,69].

### 2.2. Fluorescent Proteins

A wide variety of fluorescent proteins (FPs) has been engineered, with adaptations and characteristics to suit diverse applications. FPs can be pH-stable or pH-sensitive, and fast- or slow-maturing [70,71]. Some FPs have been engineered in split forms that fluoresce when the two halves are combined, in order to signal interactions between two proteins upon label complementation [28,71,72]. FPs may exist in monomeric, dimeric or tetrameric forms. The canonical green fluorescent protein (GFP), isolated from jellyfish (*Aequorea victoria*), is naturally a monomer [8,71], but many other FPs have a tendency to oligomerize [73,74], notably the red fluorophores derived from DsRed, which was isolated from coral (*Discosoma* spp.) [75]. Particularly at high local concentrations, such oligomerization may cause FP-tagged molecules to cluster artificially, giving rise to mislocalization artifacts [74,76]. Fortunately, several red FPs, such as mCherry, have been developed with improved monomeric character [73]. Additionally, if the FP concentration is very low, even proteins that tend to dimerize will have a low probability of finding a partner with which to pair.

**Figure 4:**
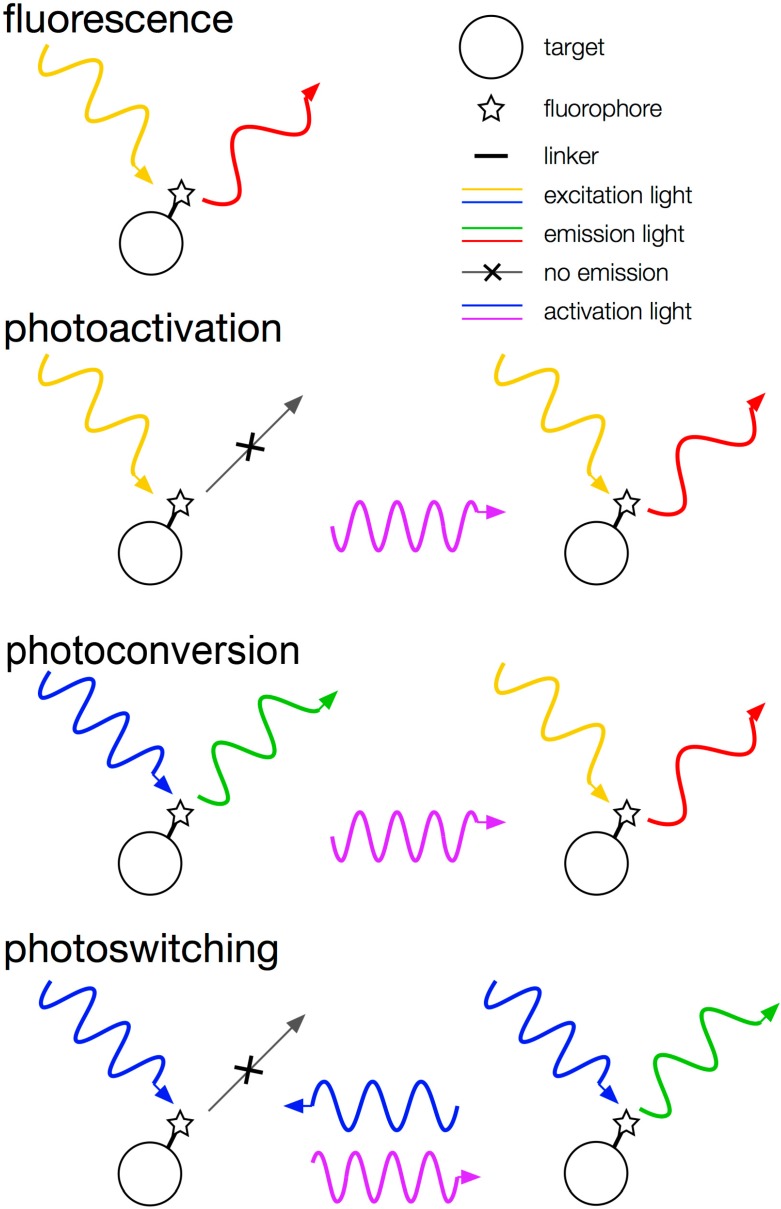
Categories of fluorescent proteins. mCherry is an example of a typical fluorescent emitter. PAmCherry is photoactivatable from off to on. Dendra2 is photoconvertible from green to red. Dronpa is reversibly photoswitchable from off to on.

In super-resolution imaging, the photophysical properties of FPs are of primary importance. FPs come in many colors, from blue to far-red. These labels can also be paired for Förster Resonance Energy Transfer (FRET) experiments [7], though the low FRET efficiency of FP pairs limits their utility for single-molecule FRET [77]. FPs can also be photoactivatable, photoconvertible, or photoswitchable (Figure 4; [70]). A *photoactivatable* fluorophore begins in a non-fluorescent “dark” state and can be switched to a fluorescent “bright” state using violet light (λ = 350–420 nm). A representative photoactivatable FP is PAmCherry, which is initially non-emissive, but after activation with violet light (405 nm), this FP absorbs yellow-green light (excitation maximum: 564 nm) and emits red light (emission maximum: 595 nm [78]). A *photoconvertible* fluorophore has two fluorescent states and can convert from the shorter-wavelength state to the longer-wavelength state. For example, Dendra2 has a “green” state (excitation maximum: 491 nm, emission maximum: 507 nm) and a “red” state (excitation maximum: 554 nm, emission maximum: 573 nm) and switches from green to red with 405-nm light [79]. A *photoswitchable* fluorophore, such as Dronpa, can *reversibly* change between a fluorescent state and a dark state. Dronpa in the bright state emits green light (emission maximum: 518 nm) upon excitation by blue light (excitation maximum: 503 nm), but intense 488-nm light can switch this molecule to a dim state. 405-nm light reactivates the fluorescent state [80].

**Figure 5:**
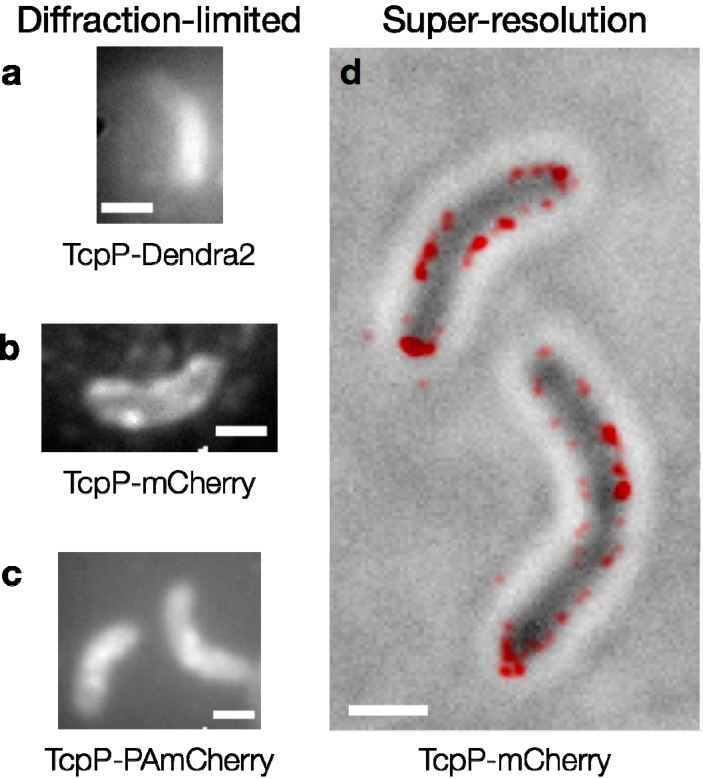
Fluorescent proteins in *V. cholerae* cells. Diffraction-limited fluorescence images of cells expressing (**a**) TcpP-Dendra2; (**b**) TcpP-mCherry and (**c**) TcpP-PAmCherry. Individual TcpP-Dendra2 molecules are not observed, and the cells in (**a**) are dimmer than those in (**b**) and (**c**). Bright foci are seen in the cells expressing TcpP-mCherry and TcpP-PAmCherry (**b**, **c**). Image acquisition times: 2–5 s; (**d**) TcpP-mCherry localizes to the cell membrane. Red: super-resolution image (based on 98 100-ms imaging frames) reconstructed from sequential localizations blurred to the 95% confidence intervals of each fit. This PALM image is plotted on top of a reverse-contrast bright field image of the cells (grey). Scale bars: 1 µm.

Genetically encoding a FP tag allows highly specific labeling of the target, but this advantage must be weighed against the poor quantum yields (e.g., 0.22 QY for mCherry [73]), low photon yields (~10^5^ photons emitted from GFP before photobleaching [81]) and large size (~25 kDa [82]) of these labels as compared to organic dyes, e.g., rhodamine 6G (0*.*95 QY, 10^6^ photons, ~0.5 kDa [83]). Moreover, not all protein fusions are stable. For example, in *V. cholerae* bacteria expressing a fusion of the membrane-bound transcription activator protein TcpP to the photoconvertible FP Dendra2, we found that the TcpP-Dendra2 fusion had a higher rate of degradation than native TcpP, though this fusion protein was still able to activate *toxT* transcription. In this same organism, other FPs, such as mCherry and PAmCherry, formed fusions with TcpP that were much more stable and thus more suitable for live-cell imaging of TcpP dynamics (Figure 5).

The desirable properties of a label depend on the goals of the experiment. In our *V. cholerae* investigations, the FP mCherry blinked (*i.e.*, switched from a bright state to a dark state) on the same timescale as our imaging (50 ms/frame), leading us to observe extremely short TcpP-mCherry trajectories, thus precluding quantitative measurements of the TcpP diffusion coefficient. On the other hand, these fluctuations in mCherry fluorescence were advantageous for creating PALM super-resolution images of all TcpP positions. By observing blinking TcpP-mCherry under 561-nm excitation, we achieved single-molecule levels of fluorescence without photoactivation simply based on the intermittent mCherry fluorescence, and we demonstrated that the TcpP-mCherry fusion is localized to the cell membrane (Figure 5(d)). This blinking behavior may be an example of temporary quenching, sometimes termed “kindling,” which is attributed to a *cis*–*trans* isomerization of the chromophore [84]. Such blinking is known to occur in mCherry and a handful of other proteins, even at low excitation powers [71,84,85].

**Figure 6:**
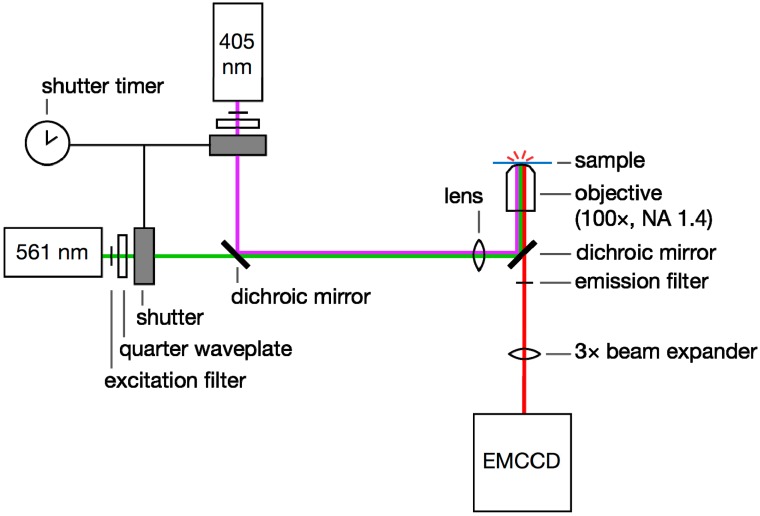
Single-molecule super-resolution imaging setup. 405- and 561-nm laser light is selected with excitation filters, circularly polarized with quarter waveplates, and controlled with a pair of shutters. The beams are coupled with a dichroic mirror. The laser beams are focused onto the back aperture of a high-NA microscope objective and the sample is excited by epi-illumination. Emitted fluorescence is filtered using an emission filter and a second dichroic. This emission is magnified with a beam expander if required and then detected on an EMCCD camera.

In our experience, the PAmCherry fluorescence was more consistent than mCherry fluorescence, and furthermore, PAmCherry emission could be controlled based on photo-activation with careful doses of 405-nm laser illumination. On the other hand, imaging PAmCherry requires a more complicated and expensive optical setup than mCherry experiments: a second (activation) laser (405 nm) must be coaligned with the imaging laser (561 nm; Figure 6). It has also been reported recently that only 4%–50% of PAmCherry molecules photoactivate into a fluorescent state [74,86]. Though this effect could not be measured in our experiments, we do not believe that inactive TcpP-PAmCherry affected our trajectory analysis, since we observe dozens of molecular trajectories per cell, and there should therefore be a sufficient number of activated molecules to represent the various modes of TcpP motion present. Certainly, however, inactivatable fluorophores could lead to underestimates in experiments designed to count molecules, and present additional challenges for satisfying the Nyquist–Shannon sampling theorem in high-resolution PALM experiments [87,88].

Another consideration when using FPs to label cellular components is the FP maturation time. Before a protein will fluoresce, it must fold properly and be oxidized to form the chromophore [8]. Typical maturation times are around 40 minutes, but depending on pH, temperature, and the specific FP, some may take multiple hours to mature [71,73,82]. Conversely, particularly fast-maturing proteins, such as the yellow FP Venus, take less than 10 minutes to mature [89]. For live-cell imaging in non-model systems, it is important to note that the chromophore formation step in GFP and DsRed derivatives requires the presence of oxygen, and therefore these traditional FPs are incompatible with obligate anaerobes.

### 2.3. Small-Molecule Dyes

Organic dye molecules have the advantage of being much brighter and more stable than fluorescent proteins and much smaller than biological molecules of interest [73,83]. The addition of a small dye molecule is unlikely to hinder protein diffusion, and with many more photons emitted before photobleaching from a dye than from a fluorescent protein, the position of an organic dye can be determined with better precision than a fluorescent protein [11]. However, small-molecule dyes are not genetically encodable and therefore must be incorporated into the cell in some other way. Not all dyes can permeate the membrane: rhodamine dyes can pass through many bacterial membranes, but only inefficiently, while sulfonated cyanine dyes are unable to cross into bacteria cells at all [70]. Endocytosis and microinjection, which are used to introduce dyes to eukaryotic cells, are not available for bacterial cells. Membrane permeabilization must be used instead, though certain permeabilization methods cause artifacts [90].

To label a specific protein with an organic dye, the protein must be engineered to incorporate a motif to which the dye molecule will bind to covalently, such as the FlAsH, HaloTag and SNAP-tag systems, among others [70,91,92,93]. In addition, since the free dye can bind nonspecifically elsewhere in the cell, most dye-labeling schemes are limited in their specificity, and all must be accompanied by washing steps to remove excess, unbound dye from the cell [70]. Very few organic dyes are photoactivatable [94], which also limits the ability to control the fluorescence. In fixed-cell imaging, reducing buffers can produce photoswitching, but most such buffers are cytotoxic [95,96].

For fixed-cell studies, fluorescently-tagged antibody labels [70] can target specific proteins. Such immunofluorescence imaging can complement live-cell studies; for example, to check the specificity of other labeling schemes [97]. However, antibodies are very bulky linkers, and, particularly when secondary antibodies are used, the fluorophore may be tens of nanometers away from the point of interest, adding to the uncertainty in the target position (Figure 3). Antibodies may also bind to multiple molecules, producing artificial clustering of the molecule of interest [70]. Despite these disadvantages, antibody labeling of bacterial surfaces or fixed cells can be a worthwhile complement to live-cell single-molecule microscopy, validating the localizations observed with other methods.

Enzymatic dye labeling schemes have been used for single-molecule microscopy in eukaryotes [70], but applications of such schemes to proteins inside live bacteria are limited to date because it is hard to get dye into these cells [98,99]. Recent examples include single extracellular membrane proteins in the *Bacteroides thetaiotaomicron* gut symbiont using both enzymatic and antibody dye labeling [97]. We have also used dye molecules to label sugars in biological systems. For example, in investigating the interaction between the *B. thetaiotaomicron* outer membrane starch utilization system (Sus) proteins and the carbohydrate amylopectin, which the Sus proteins capture and catabolize, amylopectin was labeled with AlexaFluor 488 and the SusG protein was fused to the Halo enzyme for labeling with a fluorescent HaloTag [97]. Antibody labels interfere with the starch–Sus protein interaction, so these small-molecule dyes were well-suited to these investigations.

### 2.4. Other Labeling Schemes

Several alternatives to fluorescent proteins and small-molecule dyes are being developed for single-molecule imaging in live bacteria. One exciting avenue is the incorporation of unnatural amino acids (UAAs [100]) into target proteins. Like fluorescent protein fusions, UAAs can be highly specific, genetically encodable handles. The desired UAA is encoded by a nonsense (“amber”) stop codon, which is recognized by an orthogonal tRNA–tRNA synthetase pair. Due to their small size, UAAs can be minimally perturbative, though the protein modification site must be selected to avoid truncation at the modification site [69,100].

The fluorescent coumarin amino acid (emission λ_max_ = 464 nm [101]) has been used to label the chaperonin GroEL in *E. coli* FRAP studies [100]. Other fluorescent amino acids with a variety of fluorescent properties have also been synthesized [102]. An alternative strategy to using intrinsically fluorescent UAAs is to use UAAs to incorporate functional groups (e.g., ketones, azides or alkynes) into a protein, enabling specific, covalent attachment of organic dyes through bio-orthogonal reactions [69,103,104,105,106]. A live cell-compatible demonstration of the use of UAAs and click chemistry for single-molecule microscopy has recently been reported [107]; this may provide a promising alternative to fluorescent protein labeling. Click chemistry has also been used to achieve high-density labelling of cellular DNA and RNA for super-resolution imaging [107,108].

Quantum dots and nanoparticles have also been used for labeling proteins, though rarely in super-resolution studies [64,109]. Although only nanometers in size, they are quite large compared to the labels described above (Figure 1c [64,110]). Nanoparticles are also difficult to get into cells without endocytosis, so their use in bacteria is are generally restricted to studies of the outer membrane and cell surface [64,109,111]. Because diffusion is inversely related to particle size, these nanoscale labels may slow the dynamics of the molecules whose motions they report [64,65]. Still, the main advantage of quantum dots and nanoparticles is that they have much longer photobleaching lifetimes than fluorescent proteins or small-molecule dyes, allowing longer-term observations, though quantum dots are also known to blink [9,112].

## 3. Sample Considerations

### 3.1. Achieving Single-Molecule Levels of Fluorescence

**Figure 7:**
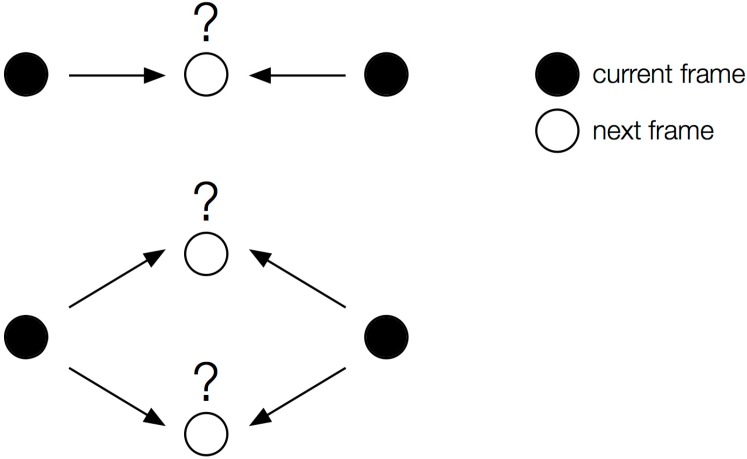
Single-molecule tracking requires low fluorophore densities. If too many localizations occur in proximity to one another, the tracking algorithm may not be able to correctly assign molecular trajectories.

Because single-molecule localization depends on detecting isolated emitters, most fitting algorithms can handle only one molecule per diffraction-limited area (~0.25 µm^2^) at a time [113]. Thus, if the fluorophore density is too high, individual molecules cannot be resolved. For bacteria like *V. cholerae*, which are about 2 µm long and 0.6 µm in diameter [37], this limit is about five fluorophores at a time per cell in ideal conditions. Data processing schemes, including multi-fluorophore fitting [113] and successive frame subtraction (e.g., Single-Molecule High-Resolution Imaging with Photobleaching, or SHRImP [20]), have been developed to improve single-molecule detectability despite high labeling densities. Still, low fluorophore density is desirable, especially if one is interested in determining diffusion dynamics based on single-molecule tracking since as fluorophore density increases, the likelihood of intersecting molecular trajectories increases, introducing error into single-molecule tracking algorithms and thus producing less reliable diffusion coefficient calculations (Figure 7).

For a genetically-encoded tag, there are several ways to control fluorescence density. In some cases, the protein of interest has a low native expression level [114]. The copy number of proteins ectopically expressed from inducible promoters can also be restricted [39,115]. For example, in our studies in *V. cholerae* of a ToxR-mCitrine protein fusion controlled by an isopropyl β-d-1-thiogalactopyranoside (IPTG)-inducible promoter, we have found that even in the absence of IPTG, promoter leakage gives rise to protein expression levels that are too high for single-molecule imaging, but glucose can repress expression enough to achieve single-molecule concentrations of this protein. Sometimes when too many fusion proteins are expressed in a single bacterial cell, partial bleaching of the sample can decrease the concentration of emissive fluorescent proteins to achieve single-molecule levels of fluorescence [32]. Still, the best control is attained when photoactivation, photoswitching, or photoconversion of fluorescent proteins permits detection of small subsets of these fluorophores at a given time [62].

In Δ*tcpP V. cholerae*, we expressed approximately 10–40 copies of TcpP-PAmCherry from an arabinose-inducible promoter by incubating these mutant cells in 0.10% arabinose in minimal media for 3 h at 30 °C. The photoactivatable PAmCherry was initially dark, and a 70-ms 0.006–0.2 µW/µm^2^ dose of 405-nm laser light rendered 1–4 PAmCherry molecules fluorescent per cell. As discussed above, this activation laser can be circumvented entirely in the case of blinking fluorescent proteins. For example, when we expressed 10–20 copies of TcpP-mCherry in Δ*tcpP* *V. cholerae* under the same conditions, the cells initially had too many fluorescing molecules for single-molecule imaging. After 2–3 minutes of photobleaching with 0.2–0.3 µW/µm^2^ 561-nm laser illumination, only about eight fluorescent mCherry molecules remained and the blinking dynamics of these probes during imaging permitted super-resolution imaging (Figure 5d).

### 3.2. Minimizing Cell Stress

Live cells pose additional challenges for single-molecule imaging because the integrity of the samples must be assured at all times. It is critical to prevent unnecessary stress to the cells. First, it is important to keep the cells in an appropriate extracellular environment: supplied with the necessary nutrients for continued growth and metabolic activity. The metabolic needs of each particular organism must be considered. In particular, to image live obligate anaerobes like *B. thetaiotaomicron*, we find it necessary to deaerate the buffer, adding reducing agents such as cysteine and sealing samples with epoxy before removal from the anaerobic growth chamber [116]. At the same time, motile cells must be immobilized to provide a stationary frame of reference for single-molecule imaging. Preparing 1%–2% agarose gel in cell media yields 100–200 nm pores [117], which provide sufficient surface roughness to immobilize cells deposited on these agarose pads during data capture while also maintaining a moist environment [62].

**Figure 8:**
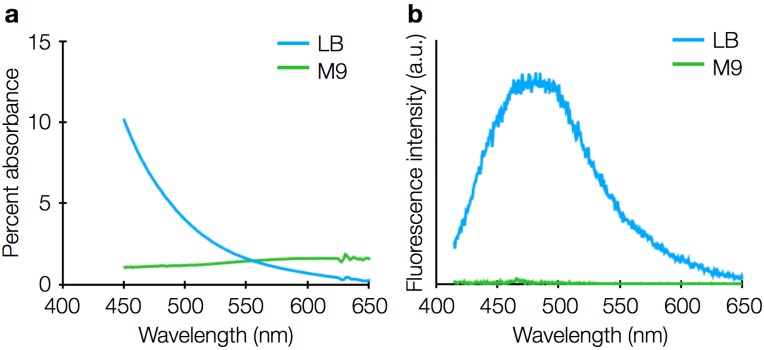
(**a**) Absorption spectra and (**b**) fluorescence emission spectra of LB rich media (blue) and M9 minimal media (green). The fluorescence emission spectrum was acquired with 405-nm excitation. Both LB and M9 have negligible fluorescence for 561-nm excitation.

**Figure 9:**
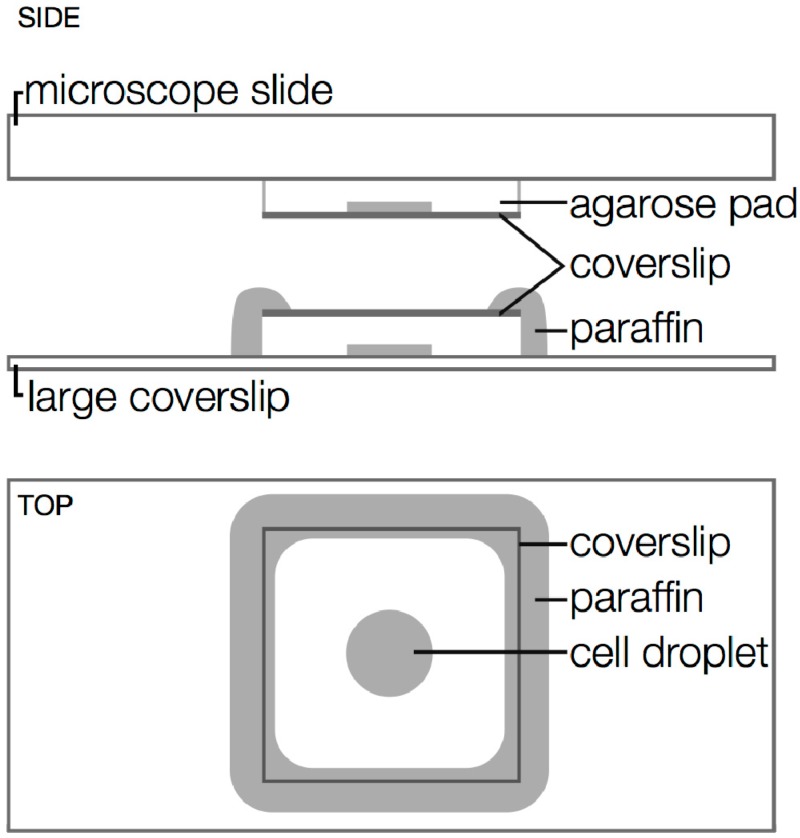
Microscopy sample geometry. Top: unsealed sample on a microscope slide. Middle and Bottom: sample sealed with paraffin wax. Side views are oriented as in an inverted microscope setup (objective at bottom).

For *V. cholerae* experiments, we determined that 2% wt/vol agarose in M9 minimal medium was sufficient to immobilize bacteria for live-cell imaging. Minimal media are preferred because rich media, such as LB medium, can add background fluorescence and increase cellular autofluorescence (Figure 8), though we have found that single-molecule imaging of intracellular fluorescent proteins is still possible in *V. cholerae* grown in LB, and that indeed that the nutrient-rich TYG media is preferred for *B. thetaiotaomicron* because it reduces cell stress. For imaging experiments lasting longer than an hour, samples may be sealed with paraffin wax or epoxy to prevent desiccation of the agarose pad (Figure 9), though sample sealing reduces oxygen delivery and is therefore harmful for obligate aerobes.

In our experiments, 1 mL aliquots of *V. cholerae* culture in M9 were centrifuged for 30 s at 30,000× *g*, washed with 1 mL warm M9, and centrifuged again. The supernatant was removed, and the cell pellet was resuspended in a minimum volume (<150 µL) of residual supernatant. A 2 µL droplet of cells was placed on a prepared agarose pad (2% agarose gel in M9) and covered with a coverslip (Figure 9). A short (5–10 min) air-drying step before subsequent sealing ensures that samples are not too wet. Without this air drying step, we have found that *V. cholerae* cells remain mobile despite the agarose gel surface roughness. In particularly wet samples, we observed *V. cholerae* cells swimming rapidly in all directions or spinning in place, presumably when caught on the agarose surface. Alternatively, microfluidic devices may be used to hold cells in place and to keep them nourished during longer or more complex experiments [118,119], and algorithms for handling single-molecule imaging within moving cells are being developed [63].

The activation and excitation lasers used may also cause cell stress. Because cells absorb violet light strongly, when photoactivating fluorophores in live cells using UV or violet light (λ = 350–450 nm), one must minimize laser powers and exposure times to avoid damaging the cells. At high power densities, even visible radiation can generate reactive species that can damage or kill cells [118,120,121]. Wagner *et al.* found that 633-nm excitation at 400 J/cm^2^ was sufficient to impact colony formation of U373-MG glioblastoma cells [122]. Emission from the fluorescent proteins themselves has a much lower power than the laser irradiation and is therefore a negligible source of cell stress. In our experiments imaging proteins in *V. cholerae*, we used low laser doses for both the activation (405 nm; 0.006–0.2 µW/µm^2^ for 50–100 ms) and the excitation (561 nm; 0.2–0.3 µW/µm^2^ for several minutes) of PA-mCherry fluorescence. One can also verify cell viability after microscopy with live/dead staining, by watching for cell division on the microscope coverslip, or by culturing cells from the sample after the microscopy experiment [39].

### 3.3. Drift

Even when the cells are stationary, the microscope stage may drift noticeably over time, either in plane or out of focus (*i.e.*, laterally or axially). If the lateral drift is slow, it can be neglected for single-molecule tracking experiments, which measure relative positions (steps) between consecutive frames, but in super-resolution PALM or STORM experiments, where the positions of molecules are recorded over a longer time, correcting for stage drift is essential [123]. Fiducial markers on the sample, such as quantum dots or fluorescent beads, can be used in post-processing to register imaging frames onto a common frame of reference [3]. Though quantum dots are small and bright, their frequent blinking [112] can complicate the image alignment process. Fluorescent beads—polymer microspheres stained with small-molecule dyes—can also be used and are commercially available in several colors as well as in multicolor compilations (e.g., TetraSpeck microspheres from Life Technologies [3]). These beads are larger than quantum dots (*>*100 nm in diameter), but because their signals come from many fluorophores, they are unlikely to blink. Unfortunately, we have found that these fluorescent beads release dye molecules into their surroundings; these dyes will adsorb to the cell and agarose pad surfaces and might even be taken up by the cells. This has led to increased background fluorescence and false single-molecule detections.

While lateral drift can be corrected in post-processing as described above, in typical two-dimensional single-molecule experiments, it is more difficult to compensate for focus drift. Stage drift of this type can be corrected manually, with fine focus or a piezoelectric objective positioner, or automatically, by using a piezo stage controlled by a feedback loop. Several labs have taken the automatic focus control one step farther to achieve three-dimensional tracking of nanoparticles and viruses in eukaryotic cells [124,125,126].

### 3.4. Sources of Background

Controlling background fluorescence is important in single-molecule imaging because the individual fluorophores have small signals that are easily overwhelmed [67]. Significant autofluorescence due to flavins in the cell is excited by blue and green lasers [127]. Using red fluorophores with appropriate emission filters can reduce the observed background. Our preferred label for tracking single proteins in live *V. cholerae* is PAmCherry1 (absorption λ_max_ = 564 nm, emission λ_max_ = 595 nm); after photoactivation, we excite this fluorescent protein with a 561-nm laser and filter out scattered laser light by approximately twelve orders of magnitude with a combination of a 580-nm long-pass dichroic mirror and a 580-nm long-pass filter (Figure 6).

Microscopy experiments were carried out at room temperature on an inverted wide-field epifluorescence microscope (Olympus IX71) using a 100× 1.40 NA oil immersion objective (Zeiss Immersol 518F immersion oil), a Semprex micrometer stage and a PIFOC piezo objective positioner. PAmCherry was photoactivated using a Coherent Cube 405-100 laser and imaged with a Coherent Cube 560-50 laser, coupled via a dichroic filter (Semrock Di01-R405). Both laser beams were circularly polarized (Tower Optical AO15Z ¼ 556, Tower Optical AO15Z ¼ 408), and the beams were alternated with a connected pair of Uniblitz shutters. Appropriate excitation, emission and dichroic filters were used (Semrock LL01-407, Semrock LL01-561, Semrock BLP01-561, Semrock Di01-R561). Emission was collected on a Photometrics Evolve EMCCD camera with >90% quantum efficiency and pixels corresponding to 49 nm × 49 nm regions of the sample (Figure 6).

Additionally, since some percentage of photoactivatable or photoconvertible fluorescent proteins will be activated by ambient light at room temperature, we have found a “pre-bleaching” step helps to decrease PAmCherry background: the sample is exposed to the excitation laser (for 2–3 min at 0.2–0.3 µW/µm^2^) to bleach pre-activated fluorophores, cellular autofluorescence, and other background sources before the first subset of fluorescent labels is intentionally photoactivated.

### 3.5. Balancing Speed with Precision

The precision of each single-molecule localization depends on the number of photons observed [11]. When wide-field single-molecule fluorescence data are recorded on an electron multiplying charge-coupled device (EMCCD) detector, the camera frame rate can be controlled from a few ms to several seconds per imaging frame. Lengthening the integration time (*i.e.*, the image exposure time) will increase the duration of each observation and thus the detected photon count, but at a cost: over the course of a long imaging frame, the emission from moving molecules will be blurred over multiple detector pixels. Such blurring increasing the uncertainty in the position determination and obscures information about molecular dynamics. For example, a cytoplasmic protein (e.g., EYFP in the cytoplasm of *E. coli*: *D* ~ 7 µm^2^∕s [38]) can diffuse the length of a *V. cholerae* cell in approximately 100 ms, which is only twice the duration of our typical imaging frame. The emission from this labeled protein will be spread out over the whole cell (~500 pixels in our configuration) and thus be unresolvable. Increasing the excitation laser power can also increase the number of photons detected, but since the fluorophore photon yield is unchanged, the dye will bleach more rapidly under higher excitation powers, and so there is a trade-off between brightness and observable trajectory lengths. For single-molecule tracking experiments, higher laser powers severely curtail the information content of a tracking experiment [128].

We imaged TcpP-PAmCherry molecules in *V. cholerae* at three different integration times: 20 ms, 50 ms, and 100 ms. In the 100-ms frames, foci were brighter, but fast motions were lost. At an integration time of 20 ms per frame, we could detect faster diffusion, but this short imaging time required higher laser powers to provide sufficient photons per imaging frame, and so the fluorophores bleached faster. In these studies of the inner-membrane-bound TcpP protein, we therefore found 50-ms frames to be the best compromise between temporal and spatial resolution.

Stroboscopic illumination can give rise to very short effective image integration times and overcome the limited time resolution of EMCCD detectors (maximum frame rate ~100 Hz). Xie *et al.* adapted such high-speed photography tricks for single-molecule imaging to track the very rapid motions of soluble cytoplasmic proteins in *E. coli* [1,67]. By using very short (*<*0.5 ms) bursts of very intense (300 W/cm^2^) excitation light coupled with long acquisition times, these experiments achieved single-molecule tracking with sub-millisecond time resolution. This stroboscopic microscopy method was applied to the single-molecule detection of gene expression products that diffused too fast to be localized with conventional illumination.

## 4. Analysis Methods

### 4.1. Localization

Single-molecule microscopy images are analyzed to determine the position of each molecule with nanometer-scale precision. In each imaging frame, the diffraction-limited intensity distribution (point-spread function) is fit to a model function (typically a two-dimensional Gaussian function), and the dye position is given by the center of the fitting function. For stationary emitters, the localization uncertainty can be determined by fitting multiple successive images, while for mobile emitters, the localization uncertainty must be estimated from a single image, for example from the 95% confidence interval of the fit [62]. These position data can then be used to construct super-resolution maps of the fluorescently labeled molecules, to form single-molecule trajectories, and to calculate dynamic information such as instantaneous velocities and the diffusion coefficient.

### 4.2. Single-Particle Tracking

**Figure 10:**
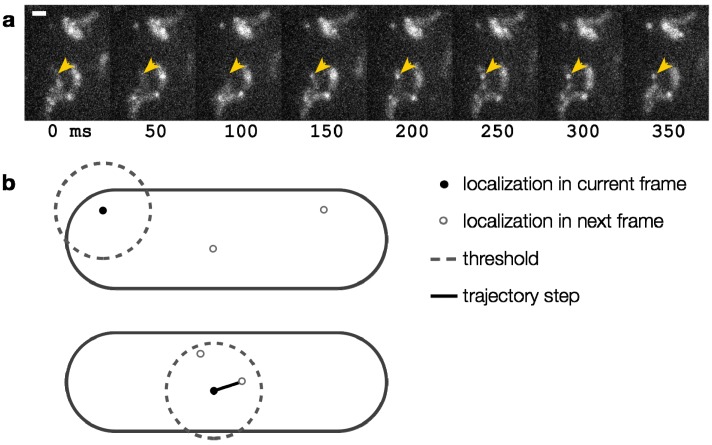
(**a**) Time-lapse images of diffusing TcpP-PAmCherry molecules in live *V. cholerae* cells. The arrowhead highlights one molecule, which turns on in the fourth panel and moves inside the cell. Each panel is 50 ms. Scale bar: 1 µm; (**b**) Implementing a step size threshold (dashed line). Top: The black molecule cannot be connected to either molecular position in the next frame because they are both beyond the maximum step size. Bottom: If more than one localization is within the threshold, the nearest is chosen.

The common metric of Brownian motion is the diffusion coefficient, *D* [129]. To determine *D* for a particular molecule, individual localizations are connected from frame to frame to form single-molecule trajectories (Figure 10a). The simplest method is a nearest-neighbor connection: two molecules located nearest to each other in consecutive frames are connected to form a trajectory. A maximum step size threshold may be used to prevent unphysical connections (Figure 10b). Unfortunately, it is difficult to choose an appropriate threshold that avoids nonsensical connections while allowing occasional large motions. Additionally, if the fluorophore density is too high, trajectories may cross, making track assignment untrustworthy (Figure 7). The main disadvantage of tracking single molecules in live bacteria cells is that the data content is limited since the trajectories are finite: tracks terminate abruptly upon fluorophore blinking and photobleaching.

We chose our threshold of 300 nm per 50-ms frame (that is, consecutive localizations more than 300 nm apart were considered to be distinct molecules) for tracks of TcpP-PAmCherry in *V. cholerae* by running the tracking algorithm without a step size limit in place and then examining the histogram of step sizes. Most steps were smaller than 150 nm, though the distribution tail reached to ~300 nm. We then ran the tracking algorithm a second time with the 300-nm threshold in place to filter out nonsensical connections, such as when a putative track was formed between different molecules at opposite ends of the cell [46].

A number of algorithms have been developed to handle merging, splitting and crossing tracks, as well as blinking [130,131,132]. Global fitting schemes have also been used, in which the most likely connections are found for all localizations simultaneously, rather than sequentially [130]. For our studies of TcpP diffusion in *V. cholerae*, we used the simplest nearest-neighbor algorithm and relied on low fluorophore concentrations to prevent tracks from crossing. We did not allow blinking in trajectories because we could not be confident that the putative steps taken during blinking frames connected the same molecule. For single-frame steps, our step size threshold was 300 nm, approximately the cell radius; if a single dark frame is allowed for blinking molecules, this two-frame step size limit (600 nm) is a large fraction of the cell length.

Alternatively, one can skip particle tracking altogether and use correlation analysis over time to extract diffusion coefficients. Two such methods are Particle Image Correlation Spectroscopy (PICS) and Spatio-Temporal Image Correlation Spectroscopy (STICS) [133,134,135]. The former measures diffusion coefficients based on correlation of sequential particle localization maps, while the latter forgoes single-molecule localization and determines diffusion coefficients by correlating the raw data of the imaging frames themselves. These two methods are sensitive for even fast-moving dyes or high fluorophore concentrations, though neither provides a map of single-molecule trajectories, which may be useful for determining the spatial dependence of molecular motion.

### 4.3. Mean Squared Displacement Analysis

Whether successive localizations are connected as tracks or motion is discerned from correlation, translating molecular displacements into a diffusion coefficient requires a model. There are several models for diffusion; the simplest is Brownian motion, in which the diffusion coefficient of a moving molecule is proportional to the slope of its mean squared displacement (MSD, ⟨*r*^2^⟩) curve, plotted as a function of time-lag, *τ*:

⟨*r*^2^⟩ = 2*nDτ* (1)

where *n* is the number of dimensions and *D* is the diffusion coefficient [136]. Other models exist for anomalous, directed and confined motions [129]. Though it is tempting to categorize diffusive behavior based on the shape of single-trajectory MSD curves, it is important to remember that the stochastic nature of Brownian diffusion can make molecular motion appear confined or anomalous when it is in fact not, particularly for tracks of finite length [137].

Short trajectories, which are very common in live-cell microscopy, can be very noisy [129]. For MSD analysis, only points in the first 50%–60% of time-lags are plotted because MSD values at larger values are averages drawn from few points and therefore have large errors [129,138]. This commonly used threshold is somewhat arbitrary [139], however. A statistically rigorous method for determining the optimum number of points to use when fitting MSD curves has been proposed instead [139]. Based on this method and our minimum trajectory length threshold of 10 frames, we calculate diffusion coefficients for fluorescent proteins in live cells using the first four points of each MSD curve.

**Figure 11:**
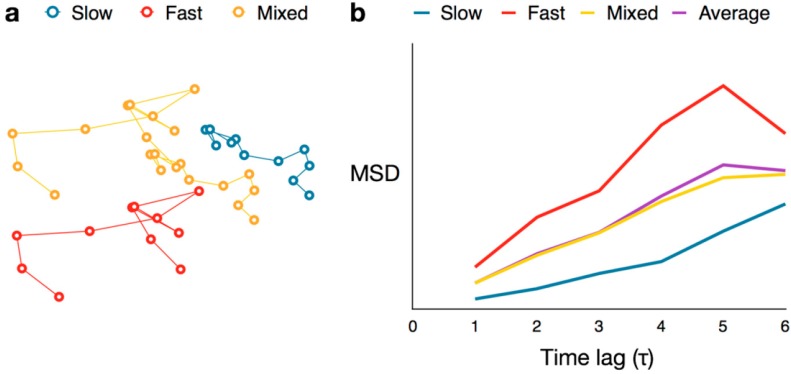
MSD averaging effect. (**a**) Three simulated trajectories for molecules exhibiting fast diffusion (red) and slow diffusion (blue), and a combination of the two (yellow), respectively; (**b**) MSD curves for the three trajectories in (**a**), as well as the curve for the mean (purple) of the fast and slow MSDs.

The major drawback of single-molecule mean squared displacement analysis is that it assumes each of the individual molecules exhibits homogeneous motion. For trajectories in which molecules change behavior, e.g., a protein slows down as it binds its target—simply extracting *D* from the slope of the MSD curves assigns diffusive roles that reflect some average behavior. Indeed, the MSD curve of a molecule that diffuses rapidly at first and then slows down is nearly indistinguishable from the average of the MSD curves of two molecules: one which diffuses rapidly and the second which has slow diffusion (Figure 11).

### 4.4. Cumulative Probability Distribution Analysis

An analytical tool that considers all steps independently of the trajectories they come from can include models for heterogeneous motion and overcome the limitations of MSD analysis described above. One such method is to use the cumulative probability distribution (CPD) of the step sizes. For two-dimensional motion, this CPD describes the probability of a molecule staying within an area defined by a radius, *r*, given the localization accuracy, *σ*, during a given time-lag, *τ* [140]. Analyzing the step size CPD by fitting it to a multi-component model (Equation (2)) provides a framework for considering heterogeneous mixtures of molecular populations [4]. This model allows for multiple populations of molecules, each with a different diffusion coefficient, by including one exponential term per population of molecules:

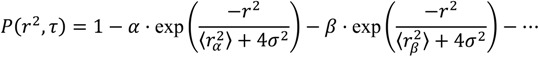
(2)


Fitting the step size CPD to this model gives the fraction of molecules in each population (*α*, *β*, *etc.*, where *α + β + ⋯* = 1), as well as the MSD for each population at each time-lag (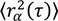, 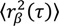, *etc.*). The MSD values are then plotted as in single-molecule MSD analysis, and the diffusion coefficient for each population is calculated from the slopes of these curves, as described above (Equation (1)). For both single-molecule MSD and CPD analysis of TcpP diffusion in *V. cholerae*, we included only trajectories with at least 10 frames, removing very noisy datasets from consideration.

**Figure 12:**
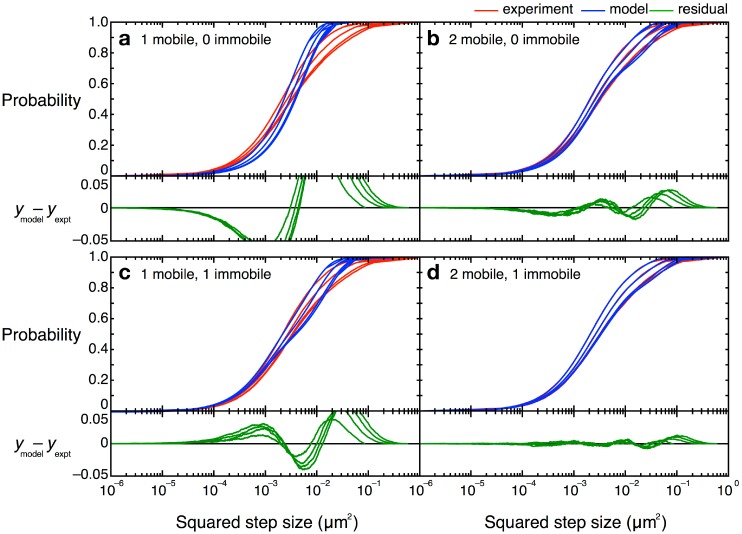
CPD curves and alternative models for TcpP-PAmCherry diffusion. Experimental data are shown in red and best fits for each of the given models are shown in blue. Curves for the first four time lags (50–200 ms) are plotted left to right. Residuals are shown in green. (**a**–**c**) Models with fewer than three terms did not describe the data well; (**d**) A model with two mobile terms and one immobile term described the CPD data best. Figure from reference [46].

In our studies of TcpP-PAmCherry diffusion in *V. cholerae*, we found a three-term model described the data the best, with one “fast” population, one “slow” population, and one population of molecules that were immobile (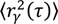 = 0 µm^2^*∕*s) within our resolution (*σ* = 30 nm). Simpler models, with only one or two terms, did not describe the data well (Figure 12). Unfortunately, though the CPD analysis could reveal the system heterogeneity, this stepwise analysis does not permit individual proteins to be classified into a specific population. The best-fit model allowed us to determine that, at any given time, 22% of TcpP-PAmCherry molecules in a Δ*tcpP* TcpP-PAmCherry strain are in the immobile population, but the aggregation of all trajectory data prevents us from determining which specific molecules are immobilized, or to which trajectories they belong. On the other hand, single-molecule MSD analysis could not give us such clear information about the population heterogeneity.

### 4.5. Curvature Challenges

**Figure 13:**
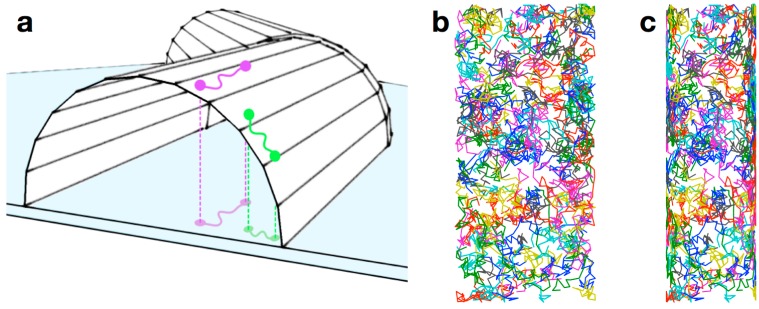
Effect of cell curvature on membrane-bound trajectories. (**a**) The purple and green paths are the same length on the surface of the cell, but the green path appears shorter than the purple path when projected onto the imaging plane (represented by the blue surface) due to the curvature of the cell membrane; (**b**) 200 two-dimensional random walks on the surface of a cylinder; (**c**) A projection of the walks in (**b**) onto a simulated two-dimensional detector shows a large degree of distortion at the edges of the projection due to the curved nature of the cell membrane. Figure from reference [46].

Bacteria are small enough (typically 0.5–1 µm in diameter [36]) to fit entirely within the focal depth of even a high-NA light microscope (0.703 µm [141]). However, though bacteria are thin, they are not two-dimensional; the actual locations and motions of molecules inside a bacterium may appear distorted in two-dimensional images. For example, a fluorescent molecule moving perpendicular to the focal plane through the cytoplasm will appear stationary. Even the two-dimensional motion of a membrane-bound protein is distorted when projected onto the imaging plane (Figure 13a). Motion parallel to the short axis of the cell, especially near the edge, appears slower than movement along the long axis of the cell, leading to an underestimation of the speed—and a concomitant underestimation of the diffusion coefficient—of the protein [4,39].

To examine the effect of membrane curvature on our membrane protein tracking results, we modeled a random walk on the upper surface of a cylinder with the same dimensions as *V. cholerae* (*i.e.*, 600 nm diameter and 2 µm length). In our model, the molecule moved randomly on a three-dimensional cylindrical surface; this trajectory was then projected onto a second two-dimensional surface to simulate detection by the EMCCD (Figure 13b,c). In this way, we compared random motion in real space to the detected motion on a plane. The two-dimensional projection clearly shows distortion due to the curvature of the membrane (Figure 13c). To quantify this distortion, we simulated 200 molecules taking steps chosen from a distribution corresponding to a diffusion coefficient of 0.066 µm2/s. The best-fit CPD model for the projection of the walk had two terms: a large (~95%) mobile population (with an apparent diffusion coefficient of 0.057 µm^2^/s) and a small (4%–6%) immobile population. The immobile population is entirely attributable to curvature artifacts, while the calculated fast diffusion coefficient demonstrates that the curvature leads to an underestimation of the true diffusion. In our experimental data for TcpP-PAmCherry diffusion in *V. cholerae*, an immobile population was present in three cell strains, but it was significantly larger (10%–20%) than could be attributed to the curvature artifact alone. We concluded that there does in fact exist a nontrivial population of TcpP molecules that are immobile within our 30-nm resolution.

There are several ways to account for the effects of imaging inherently three-dimensional motion on a two-dimensional plane. For example, one can explicitly take the shape of the cell into account and use simulations to find a conversion factor back to the actual diffusion coefficient [39]. Alternatively, one can manipulate the point-spread function to obtain information about the position of the molecule in the *z* axis (*i.e.*, perpendicular to the imaging plane). In astigmatic imaging, a cylindrical lens is inserted into the emitted light path, distorting the symmetric point spread function (PSF) of dipole emission into an elliptical point-spread function (PSF) whose aspect ratio depends on the *z* position [142,143]. Another example of emission-beam engineering is the creation of a double-helix PSF. By manipulating the emission beam in Fourier space, the standard PSF is transformed into two diffraction-limited spots, and the angle of the two points changes as a function of *z*-position [144,145]. Additional approaches to three-dimensional imaging are reviewed in reference [146].

## 5. Conclusions

There are many obstacles to consider when designing a single-molecule fluorescence experiment in live cells, including fluorophore choice, labeling scheme, cell viability, sample drift, and membrane curvature, but these are not insurmountable. Indeed, several groups have used super-resolution localization microscopy and single-molecule tracking to study structure, dynamics and molecular-scale interactions in live bacteria, yielding new insight into the structural proteins FtsZ and MreB in *C. crescentus* and *E. coli* [115,143,147], gene transcription and transcription-factor DNA looping in *E. coli* [67,68,148], nucleoid-associated proteins in both *C. crescentus* and *E. coli* [44,149], and facilitated diffusion of the *lac* repressor in *E. coli* [150]. We have extended these methods beyond model systems to examine the starch utilization system in *B. thetaiotaomicron* [97] and the pathogenic pathway in live *V. cholerae* [46]. As single-molecule techniques become more accessible to non-experts, we expect these real-time super-resolution methods to be applied to a greater diversity of systems and to reveal new insights about life at the molecular scale.

## References

[1] Xie X.S., Choi P.J., Li G.W., Lee N.K., Lia G. (2008). Single-molecule approach to molecular biology in living bacterial cells. Annu. Rev. Biophys..

[2] Huang B., Bates M., Zhuang X.W. (2009). Super-resolution fluorescence microscopy. Annu. Rev. Biochem..

[3] Biteen J.S., Moerner W.E. (2010). Single-molecule and superresolution imaging in live bacteria cells. Cold Spring Harb. Perspect. Biol..

[4] Van den Wildenberg S.M.J.L., Bollen Y.J.M., Peterman E.J.G. (2011). How to quantify protein diffusion in the bacterial membrane. Biopolymers.

[5] Abbe E. (1873). Beiträge zur theorie des mikroskops und der mikroskopischen wahrnehmung. Archiv. Mikrosk. Anat..

[6] Chiu W., Baker M.L., Jiang W., Dougherty M., Schmid M.F. (2005). Electron cryomicroscopy of biological machines at subnanometer resolution. Structure.

[7] Förster T. (1948). Zwischenmolekulare energiewanderung und fluoreszenz. Ann. Phys..

[8] Ormö M., Cubitt A.B., Kallio K., Gross L.A., Tsien R.Y., Remington S.J. (1996). Crystal structure of the *Aequorea victoria* green fluorescent protein. Science.

[9] Michalet X., Pinaud F.F., Bentolila L.A., Tsay J.M., Doose S., Li J.J., Sundaresan G., Wu A.M., Gambhir S.S., Weiss S. (2005). Quantum dots for live cells, *in vivo* imaging, and diagnostics. Science.

[10] Chen Y., Cai J., Xu Q., Chen Z.W. (2004). Atomic force bio-analytics of polymerization and aggregation of phycoerythrin-conjugated immunoglobulin G molecules. Mol. Immunol..

[11] Thompson R.E., Larson D.R., Webb W.W. (2002). Precise nanometer localization analysis for individual fluorescent probes. Biophys. J..

[12] Betzig E., Patterson G.H., Sougrat R., Lindwasser O.W., Olenych S., Bonifacino J.S., Davidson M.W., Lippincott-Schwartz J., Hess H.F. (2006). Imaging intracellular fluorescent proteins at nanometer resolution. Science.

[13] Hess S.T., Girirajan T.P.K., Mason M.D. (2006). Ultra-high resolution imaging by fluorescence photoactivation localization microscopy. Biophys. J..

[14] Rust M.J., Bates M., Zhuang X. (2006). Sub-diffraction-limit imaging by stochastic optical reconstruction microscopy (STORM). Nat. Methods.

[15] Zhu L., Zhang W., Elnatan D., Huang B. (2012). Faster STORM using compressed sensing. Nat Methods.

[16] Yildiz A., Forkey J.N., McKinner S.A., Ha T., Goldman Y.E., Selvin P.R. (2003). Myosin V walks hand-over-hand: Single fluorophore imaging with 1.5-nm localization. Science.

[17] Hell S.W., Wichmann J. (1994). Breaking the diffraction resolution limit by stimulated emission: Stimulated-emission-depletion fluorescence microscopy. Opt. Lett..

[18] Gustafsson M.G.L. (2005). Nonlinear structured-illumination microscopy: Wide-field fluorescence imaging with theoretically unlimited resolution. Proc. Natl. Acad. Sci. USA.

[19] Hell S.W. (2007). Far-field optical nanoscopy. Science.

[20] Gordon M.P., Ha T., Selvin P.R. (2004). Single-molecule high-resolution imaging with photobleaching. Proc. Natl. Acad. Sci. USA.

[21] Qu X., Wu D., Mets L., Scherer N.F. (2004). Nanometer-localized multiple single-molecule fluorescence microscopy. Proc. Natl. Acad. Sci. USA.

[22] Churchman L.S., Oekten Z., Rock R.S., Dawson J.F., Spudich J.A. (2005). Single molecule high-resolution colocalization of Cy3 and Cy5 attached to macromolecules measures intramolecular distances through time. Proc. Natl. Acad. Sci. USA.

[23] Lidke K., Rieger B., Jovin T., Heintzmann R. (2005). Superresolution by localization of quantum dots using blinking statistics. Opt. Express.

[24] Sharonov A., Hochstrasser R.M. (2006). Wide-field subdiffraction imaging by accumulated binding of diffusing probes. Proc. Natl. Acad. Sci. USA.

[25] Egner A., Geisler C., von Middendorff C., Bock H., Wenzel D., Medda R., Andresen M., Stiel A.C., Jakobs S., Eggeling C. (2007). Fluorescence nanoscopy in whole cells by asynchronous localization of photoswitching emitters. Biophys. J..

[26] Heilemann M., van de Linde S., Schüttpelz M., Kasper R., Seefeldt B., Mukherjee A., Tinnefeld P., Sauer M. (2008). Subdiffraction-resolution fluorescence imaging with conventional fluorescent probes. Angew. Chem. Int. Ed..

[27] Giannone G., Hosy E., Levet F., Constals A., Schulze K., Sobolevsky A.I., Rosconi M.P., Gouaux E., Tampé R., Choquet D. (2010). Dynamic superresolution imaging of endogenous proteins on living cells at ultra-high density. Biophys. J..

[28] Pinaud F., Dahan M. (2011). Targeting and imaging single biomolecules in living cells by complementation-activated light microscopy with split-fluorescent proteins. Proc. Natl. Acad. Sci. USA.

[29] Schoen I., Ries J., Klotzsch E., Ewers H., Vogel V. (2011). Binding-activated localization microscopy of DNA structures. Nano Lett..

[30] Lew M.D., Lee S.F., Ptacin J.L., Lee M.K., Tweig R.J., Shapiro L., Moerner W.E. (2011). Three-dimensional superresolution colocalization of intracellular protein superstructures and the cell surface in live *Caulobacter crescentus*. Proc. Natl. Acad. Sci. USA.

[31] Burnette D.T., Sengupta P., Dai Y., Lippincott-Schwartz J., Kachar B. (2011). Bleaching/blinking assisted localization microscopy for superresolution imaging using standard fluorescent molecules. Proc. Natl. Acad. Sci. USA.

[32] Moerner W.E. (2012). Microscopy beyond the diffraction limit using actively controlled single molecules. J. Microsc..

[33] Appelhans T., Richter C.P., Wilkens V., Hess S.T., Piehler J., Busch K.B. (2012). Nanoscale organization of mitochondrial microcompartments revealed by combining tracking and localization microscopy. Nano Lett..

[34] Magde D., Elson E., Webb W.W. (1972). Thermodynamic fluctuations in a reacting System—Measurement by fluorescence correlation spectroscopy. Phys. Rev. Lett..

[35] Peters R., Peters J., Tews K.H., Bähr W. (1974). A microfluorimetric study of translational diffusion in erythrocyte membranes. Biochim. Biophys. Acta.

[36] Koch A.L. (1996). What size should a bacterium be? A question of scale. Annu. Rev. Microbiol..

[37] Kay B.A., Bopp C.A., Wells J.G., Wachsmuth I.K., Blake P.A., Olsvik Ø. (1994). Isolation and identification of *Vibrio cholerae* O1 from fecal specimens. Vibrio Cholerae and Cholera: Molecular to Global Perspectives.

[38] Kumar M., Mommer M.S., Sourjik V. (2010). Mobility of cytoplasmic, membrane, and DNA-binding proteins in *Escherichia coli*. Biophys. J..

[39] Deich J., Judd E.M., McAdams H.H., Moerner W.E. (2004). Visualization of the movement of single histidine kinase molecules in live *Caulobacter* cells. Proc. Natl. Acad. Sci. USA.

[40] Logan N.A. (1994). Bacterial Systematics.

[41] Rosselló-Mora R., Amann R. (2001). The species concept for prokaryotes. FEMS Microbiol. Rev..

[42] Baker H., Bloom W.L. (1948). Further studies on the gram stain. J. Bacteriol..

[43] Kuo S.C., McGrath J.L. (2000). Steps and fluctuations of listeria monocytogenes during actin-based motility. Nature.

[44] Wang W., Li G., Chen C., Xie X.S., Zhuang X. (2011). Chromosome organization by a nucleoid-associated protein in live bacteria. Science.

[45] Bakshi S., Siryaporn A., Goulian M., Weisshaar J.C. (2012). Superresolution imaging of ribosomes and RNA polymerase in live *Escherichia coli* cells. Mol. Microbiol..

[46] Haas B.L., Matson J.S., DiRita V.J., Biteen J.S. (2014). Single-molecule tracking in live *Vibrio cholerae* reveals that ToxR recruits the membrane-bound transcription activator TcpP to the *toxT* promoter.

[47] Matson J.S., Withey J.H., DiRita V.J. (2007). Regulatory networks controlling *Vibrio cholerae* virulence gene expression. Infect. Immun..

[48] Miller V.L., Taylor R.K., Mekalanos J.J. (1987). Cholera toxin transcriptional activator ToxR is a transmembrane DNA binding protein. Cell.

[49] Häse C.C., Mekalanos J.J. (1998). TcpP protein is a positive regulator of virulence gene expression in *Vibrio cholerae*. Proc. Natl. Acad. Sci. USA.

[50] Merrell D.S., Camilli A. (2000). Regulation of *Vibrio cholerae* genes required for acid tolerance by a member of the “ToxR-like” family of transcriptional regulators. J. Bacteriol..

[51] Dalia A.B., Lazinski D.W., Camilli A. (2014). Identification of a membrane-bound transcriptional regulator that links chitin and natural competence in *Vibrio cholerae*. mBio.

[52] Kolibachuk D., Greenberg E.P. (1993). The *Vibrio fischeri* luminescence gene activator LuxR is a membrane-associated protein. J. Bacteriol..

[53] Reich K.A., Schoolnik G.K. (1994). The light organ symbiont *Vibrio fischeri* possesses a homolog of the *Vibrio cholerae* transmembrane transcriptional activator ToxR. J. Bacteriol..

[54] Lin Z., Kumagai K., Baba K., Mekalanos J.J., Nishibuchi M. (1993). *Vibrio parahaemolyticus* has a homolog of the *Vibrio cholerae toxRS* operon that mediates environmentally induced regulation of the thermostable direct hemolysin gene. J. Bacteriol..

[55] Neely M.N., Dell C.L., Olson E.R. (1994). Roles of LysP and CadC in mediating the lysine requirement for acid induction of the *Escherichia coli cad* operon. J. Bacteriol..

[56] D’Elia J.N., Salyers A.A. (1996). Effect of regulatory protein levels on utilization of starch by *Bacteroides thetaiotaomicron*. J. Bacteriol..

[57] Yang Y., Isberg R.R. (1997). Transcriptional regulation of the *Yersinia pseudotuberculosis* pH 6 antigen adhesin by two envelope-associated components. Mol. Microbiol..

[58] Welch T.J., Bartlett D.H. (1998). Identification of a regulatory protein required for pressure-responsive gene expression in the deep-sea bacterium *Photobacterium* species strain SS9. Mol. Microbiol..

[59] Blanc-Potard A., Solomon F., Kayser J., Groisman E.A. (1999). The SPI-3 pathogenicity island of *Salmonella enterica*. J. Bacteriol..

[60] Lassak K., Peeters E., Wróbel S., Albers S. (2013). The one-component system ArnR: A membrane-bound activator of the crenarchaeal archaellum. Mol. Microbiol..

[61] Bates M., Huang B., Dempsey G.T., Zhuang X. (2007). Multicolor super-resolution imaging with photo-switchable fluorescent probes. Science.

[62] Biteen J.S., Thompson M.A., Tselentis N.K., Bowman G.R., Shapiro L., Moerner W.E. (2008). Super-resolution imaging in live *Caulobacter crescentus* cells using photoswitchable EYFP. Nat. Methods.

[63] Rowland D.J., Biteen J.S. (2014). Top-hat and asymmetric gaussian-based fitting functions for quantifying directional single-molecule motion. ChemPhysChem.

[64] Ritchie K., Lill Y., Sood C., Lee H., Zhang S. (2013). Single-molecule imaging in live bacteria cells. Philos. Trans. R. Soc. B.

[65] Einstein A. (1905). Über die von der molekularkinetischen theorie der wärme geforderte bewegung von in ruhenden flüssigkeiten suspendierten teilchen. Ann. Phys..

[66] Beck N.A., Krukonis E.S., DiRita V.J. (2004). TcpH influences virulence gene expression in *Vibrio cholerae* by inhibiting degradation of the transcription activator TcpP. J. Bacteriol..

[67] Yu J., Xiao J., Ren X., Lao K., Xie X.S. (2006). Probing gene expression in live cells, one protein molecule at a time. Science.

[68] Elf J., Li G.W., Xie X.S. (2007). Probing transcription factor dynamics at the single-molecule level in a living cell. Science.

[69] Chen I., Ting A. (2005). Site-specific labeling of proteins with small molecules in live cells. Curr. Opin. Biotechnol..

[70] Fernandez-Suarez M., Ting A.Y. (2008). Fluorescent probes for super-resolution imaging in living cells. Nat. Rev. Mol. Cell Biol..

[71] Chudakov D.M., Matz M.V., Lukyanov S., Lukyanov K.A. (2010). Fluorescent proteins and their applications in imaging living cells and tissues. Physiol. Rev..

[72] Hu C., Kerppola T.K. (2003). Simultaneous visualization of multiple protein interactions in living cells using multicolor fluorescence complementation analysis. Nat. Biotechnol..

[73] Shaner N.C., Campbell R.E., Steinbach P.A., Giepmans B.N.G., Palmer A.E., Tsien R.Y. (2004). Improved monomeric red, orange and yellow fluorescent proteins derived from *Discosoma* sp. red fluorescent protein. Nat. Biotechnol..

[74] Wang S., Moffitt J.R., Dempsey G.T., Xie X.S., Zhuang X. (2014). Characterization and development of photoactivatable fluorescent proteins for single-molecule-based superresolution imaging. Proc. Natl. Acad. Sci. USA.

[75] Matz M.V., Fradkov A.F., Labas Y.A., Savitsky A.P., Zaraisky A.G., Markelov M.L., Lukyanov S.A. (1999). Fluorescent proteins from nonbioluminescent anthozoa species. Nat. Biotechnol..

[76] Verkhusha V.V., Lukyanov K.A. (2004). The molecular properties and applications of anthozoa fluorescent proteins and chromoproteins. Nat. Biotechnol..

[77] Akrap N., Seidel T., Barisas B.G. (2010). Förster distances for fluorescent resonant energy transfer between mCherry and other visible fluorescent proteins. Anal. Biochem..

[78] Subach F.V., Patterson G.H., Manley S., Gillette J.M., Lippincott-Schwartz J., Verkhusha V.V. (2009). Photoactivatable mCherry for high-resolution two-color fluorescence microscopy. Nat. Methods.

[79] Gurskaya N.G., Verkhusha V.V., Shcheglov A.S., Staroverov D.B., Chepurnykh T.V., Fradkov A.F., Lukyanov S.A., Lukyanov K.A. (2006). Engineering of a monomeric green-to-red photoactivatable fluorescent protein induced by blue light. Nat. Biotechnol..

[80] Habuchi S., Ando R., Dedecker P., Verheijen W., Mizuno H., Miyawaki A., Hofkens J. (2005). Reversible single-molecule photoswitching in the GFP-like fluorescent protein dronpa. Proc. Natl. Acad. Sci. USA.

[81] Kubitscheck U., Kueckmann O., Kues T., Peters R. (2000). Imaging and tracking of single GFP molecules in solution. Biophys. J..

[82] Baird G.S., Zacharias D.A., Tsien R.Y. (2000). Biochemistry, mutagenesis, and oligomerization of DsRed, a red fluorescent protein from coral. Proc. Natl. Acad. Sci. USA.

[83] Willets K.A., Nishimura S.Y., Schuck P.J., Twieg R.J., Moerner W.E. (2005). Nonlinear optical chromophores as nanoscale emitters for single-molecule spectroscopy. Acc. Chem. Res..

[84] Chudakov D.M., Feofanov A.V., Mudrik N.N., Lukyanov S., Lukyanov K.A. (2003). Chromophore environment provides clue to kindling fluorescent protein riddle. J. Biol. Chem..

[85] Shaner N.C., Lin M.Z., McKeown M.R., Steinbach P.A., Hazelwood K.L., Davidson M.W., Tsien R.Y. (2008). Improving the photostability of bright monomeric orange and red fluorescent proteins. Nat. Methods.

[86] Durisic N., Laparra-Cuervo L., Sandoval-Álvarez Á, Borbely J.S., Lakadamyali M. (2004). Single-molecule evaluation of fluorescent protein photoactivation efficiency using an *in vivo* nanotemplate. Nat. Methods.

[87] Shannon C.E. (1949). Communication in the presence of noise. Proc. IRE.

[88] Shroff H., Galbraith C.G., Galbraith J.A., Betzig E. (2008). Live-cell photoactivated localization microscopy of nanoscale adhesion dynamics. Nat. Methods.

[89] Nagai T., Ibata K., Park E.S., Kubota M., Mikoshiba K., Miyawaki A. (2002). A variant of yellow fluorescent protein with fast and efficient maturation for cell-biological applications. Nat. Biotechnol..

[90] Sochacki K.A., Shkel I.A., Record M.T., Weisshaar J.C. (2011). Protein diffusion in the periplasm of *E. coli* under osmotic stress. Biophys. J..

[91] Griffin B.A., Adams S.R., Tsien R.Y. (1998). Specific covalent labeling of recombinant protein molecules inside live cells. Science.

[92] Los G.V., Encell L.P., McDougall M.G., Hartzell D.D., Karassina N., Zimprich C., Wood M.G., Learish R., Ohana R.F., Urh M. (2008). HaloTag: A novel protein labeling technology for cell imaging and protein analysis. ACS Chem. Biol..

[93] Keppler A., Gendreizig S., Gronemeyer T., Pick H., Vogel H., Johnsson K. (2003). A general method for the covalent labeling of fusion proteins with small molecules *in vivo*. Nat. Biotechnol..

[94] Vogelsang J., Steinhauer C., Forthmann C., Stein I.H., Person-Skegro B., Cordes T., Tinnefeld P. (2010). Make them blink: Probes for super-resolution microscopy. ChemPhysChem.

[95] Endesfelder U., Malkusch S., Flottmann B., Mondry J., Liguzinski P., Verveer P.J., Heilemann M. (2011). Chemically induced photoswitching of fluorescent probes—A general concept for super-resolution microscopy. Molecules.

[96] Henriques R., Griffiths C., Hesper Rego E., Mhlanga M.M. (2011). PALM and STORM: Unlocking live-cell super-resolution. Biopolymers.

[97] Karunatilaka K.S., Cameron E.A., Martens E.C., Koropatkin N.M., Biteen J.S. (2014). Super-resolution imaging captures carbohydrate utilization dynamics in human gut symbionts.

[98] Nicolle O., Rouillon A., Guyodo H., Tamanai-Shacoori Z., Chandad F., Meuric V., Bonnaure-Mallet M. (2010). Development of SNAP-tag-mediated live cell labeling as an alternative to GFP in *Porphyromonas gingivalis*. FEMS Immunol. Med. Microbiol..

[99] Seyfert K., Oosaka T., Yagnuma H., Ernst S., Noji H., Iino R., Börsch M. Subunit Rotation in a Single F_0_F_1_-ATP Synthase in a Living Bacterium Monitored by FRET. http://arxiv.org/ftp/arxiv/papers/1102/1102.2184.pdf.

[100] Charbon G., Wang J., Brustad E., Schultz P.G., Horwich A.L., Jacobs-Wagner C., Chapman E. (2011). Localization of GroEL determined by *in vivo* incorporation of a fluorescent amino acid. Bioorg. Med. Chem. Lett..

[101] Brun M.P., Bischoff L., Garbay C. (2004). A very short route to enantiomerically pure coumarin-bearing fluorescent amino acids. Angew. Chem. Int. Ed..

[102] Katritzky A.R., Narindoshvili T. (2009). Fluorescent amino acids: Advances in protein-extrinsic fluorophores. Org. Biomol. Chem..

[103] Chin J.W., Martin A.B., King D.S., Wang L., Schultz P.G. (2002). Addition of a photocrosslinking amino acid to the genetic code of *Escherichia coli*. Proc. Natl. Acad. Sci. USA.

[104] Deiters A., Cropp T.A., Mukherji M., Chin J.W., Anderson J.C., Schultz P.G. (2003). Adding amino acids with novel reactivity to the genetic code of *Saccharomyces cerevisiae*. J. Am. Chem. Soc..

[105] Zhang Z., Smith B.A.C., Wang L., Brock A., Cho C., Schultz P.G. (2003). A new strategy for the site-specific modification of proteins *in vivo*. Biochemistry.

[106] Grammel M., Hang H.C. (2013). Chemical reporters for biological discovery. Nat. Chem. Biol..

[107] Raulf A., Spahn C.K., Zessin P.J.M., Finan K., Bernhardt S., Heckel A., Heilemann M. (2014). Click chemistry facilitates direct labelling and super-resolution imaging of nucleic acids and proteins. RSC Adv..

[108] Heilemann M., Margeat E., Kasper R., Sauer M., Tinnefeld P. (2005). Carbocyanine dyes as efficient reversible single-molecule optical switch. J. Am. Chem. Soc..

[109] Zhang R., Rothenberg E., Fruhwirth G., Simonson P.D., Ye F., Golding I., Ng T., Lopes W., Selvin P.R. (2011). Two-photon 3D FIONA of individual quantum dots in an aqueous environment. Nano Lett..

[110] Mutavdžić D., Xu J., Thakur G., Triulzi R., Kasas S., Jeremić M., Leblanc R., Radotić K. (2011). Determination of the size of quantum dots by fluorescence spectroscopy. Analyst.

[111] Chalmers N.I., Palmer J., Robert J., Du-Thumm L., Sullivan R., Shi W., Kolenbrander P.E. (2007). Use of quantum dot luminescent probes to achieve single-cell resolution of human oral bacteria in biofilms. Appl. Environ. Microbiol..

[112] Mahler B., Spinicelli P., Buil S., Quelin X., Hermier J., Dubertret B. (2008). Towards non-blinking colloidal quantum dots. Nat. Mater..

[113] Huang F., Schwartz S.L., Byars J.M., Lidke K.A. (2011). Simultaneous multiple-emitter fitting for single molecule super-resolution imaging. Biomed. Opt. Express.

[114] Taniguchi Y., Choi P.J., Li G., Chen H., Babu M., Hearn J., Emili A., Xie X.S. Quantifying *E. coli* proteome and transcriptome with single-molecule sensitivity in single cells. Science.

[115] Kim S.Y., Gitai Z., Kinkhabwala A., Shapiro L., Moerner W.E. (2006). Single molecules of the bacterial actin MreB undergo directed treadmilling motion in *Caulobacter crescentus*. Proc. Natl. Acad. Sci. USA.

[116] Karunatilaka K.S., Coupland B.R., Cameron E.A., Martens E.C., Koropatkin N.M., Biteen J.S. (2013). Single-molecule imaging can be achieved in live obligate anaerobic bacteria. Proc. SPIE.

[117] Narayanan J., Xiong J., Liu X. (2006). Determination of agarose gel pore size: Absorbance measurements vis *a* vis other techniques. J. Phys.: Conf. Ser..

[118] Xiao J., Elf J., Li G.-W., Yu J., Xie X.S., Selvin P.R., Ha T. (2008). Imaging gene expression in living cells at the single-molecule level. Single-Molecule Techniques: A Laboratory Manual.

[119] Moolman M.C., Huang Z., Krishnan S.T., Kerssemakers J.W.J., Dekker N.H. Electron Beam Fabrication of a Microfluidic Device for Studying Submicron-Scale Bacteria. http://www.biomedcentral.com/content/pdf/1477-3155-11-12.pdf.

[120] (2013). Artifacts of light. Nat. Methods.

[121] Endesfelder U., Heilemann M. (2014). Art and artifacts in single-molecule localization microscopy: Beyond attractive images. Nat. Methods.

[122] Wagner M., Weber P., Bruns T., Strauss W.S., Wittig R., Schneckenburger H. (2010). Light dose is a limiting factor to maintain cell viability in fluorescence microscopy and single molecule detection. Int. J. Mol. Sci..

[123] Jones S.A., Shim S., He J., Zhuang X. (2011). Fast, three-dimensional super-resolution imaging of live cells. Nat. Methods.

[124] Peters I.M., de Grooth B.G., Schins J.M., Figdor C.G., Greve J. (1998). Three dimensional single-particle tracking with nanometer resolution. Rev. Sci. Instrum..

[125] Dupont A., Lamb D.C. (2011). Nanoscale three-dimensional single particle tracking. Nanoscale.

[126] Welsher K., Yang H. (2014). Multi-resolution 3D visualization of the early stages of cellular uptake of peptide-coated nanoparticles. Nat. Nanotechnol..

[127] Benson R.C., Meyer R.A., Zaruba M.E., McKhann G.M. (1979). Cellular autofluorescence–is it due to flavins?. J. Histochem. Cytochem..

[128] Michalet X., Berglund A.J. (2012). Optimal diffusion coefficient estimation in single-particle tracking. Phys. Rev. E Stat. Nonlin. Soft Matter Phys..

[129] Saxton M.J. (1997). Single-particle tracking: The distribution of diffusion coefficients. Biophys. J..

[130] Jaqaman K., Loerke D., Mettlen M., Kuwata H., Grinstein S., Schmid S.L., Danuser G. (2008). Robust single-particle tracking in live-cell time-lapse sequences. Nat. Methods.

[131] Jaqaman K., Danuser G. (2009). Computational image analysis of cellular dynamics: A case study based on particle tracking. Cold Spring Harb. Protoc..

[132] Shuang B., Byers C.P., Kisley L., Wang L., Zhao J., Morimura H., Link S., Landes C.F. (2013). Improved analysis for determining diffusion coefficients from short, single-molecule trajectories with photoblinking. Langmuir.

[133] Hebert B., Costantino S., Wiseman P.W. (2005). Spatiotemporal image correlation spectroscopy (STICS) theory, verification, and application to protein velocity mapping in living CHO cells. Biophys. J..

[134] Semrau S., Schmidt T. (2007). Particle image correlation spectroscopy (PICS): Retrieving nanometer-scale correlations from high-density single-molecule position data. Biophys. J..

[135] Di Rienzo C., Gratton E., Beltram F., Cardarelli F. (2013). Fast spatiotemporal correlation spectroscopy to determine protein lateral diffusion laws in live cell membranes. Proc. Natl. Acad. Sci. USA.

[136] Anderson C.M., Georgiou G.N., Morrison I.E., Stevenson G.V., Cherry R.J. (1992). Tracking of cell surface receptors by fluorescence digital imaging microscopy using a charge-coupled device camera. low-density lipoprotein and influenza virus receptor mobility at 4 degrees C. J. Cell Sci..

[137] Robson A., Burrage K., Leake M.C. (2013). Inferring diffusion in single live cells at the single molecule level. Philos. Trans. R. Soc. B.

[138] Qian H., Sheetz M.P., Elson E.L. (1991). Single particle tracking. Analysis of diffusion and flow in two-dimensional systems. Biophys. J..

[139] Michalet X. (2011). Mean square displacement analysis of single-particle trajectories with localization error: Brownian motion in an isotropic medium. Phys. Rev. E Stat. Nonlin. Soft Matter Phys..

[140] Schütz G.J., Schindler H., Schmidt T. (1997). Single-molecule microscopy on model membranes reveals anomalous diffusion. Biophys. J..

[141] Qiu Y., Chen X., Li Y., Zheng B., Li S., Chen W.R., Liu H. (2012). Impact of the optical depth of field on cytogenetic image quality. J. Biomed. Opt..

[142] Huang B., Wang W., Bates M., Zhuang X. (2008). Three-dimensional super-resolution imaging by stochastic optical reconstruction microscopy. Science.

[143] Biteen J.S., Goley E.D., Shapiro L., Moerner W.E. (2012). Three-dimensional super-resolution imaging of the midplane protein FtsZ in live *Caulobacter crescentus* cells using astigmatism. ChemPhysChem.

[144] Pavani S.R.P., Thompson M.A., Biteen J.S., Lord S.J., Liu N., Twieg R.J., Piestun R., Moerner W.E. (2009). Three-dimensional, single-molecule fluorescence imaging beyond the diffraction limit by using a double-helix point spread function. Proc. Natl. Acad. Sci. USA.

[145] Lee H.D., Sahl S.J., Lew M.D., Moerner W.E. (2012). The double-helix microscope super-resolves extended biological structures by localizing single blinking molecules in three dimensions with nanoscale precision. Appl. Phys. Lett..

[146] Fischer R.S., Wu Y., Kanchanawong P., Shroff H., Waterman C.M. (2011). Microscopy in 3D: A biologist’s toolbox. Trends Cell Biol..

[147] Fu G., Huang T., Buss J., Coltharp C., Hensel Z., Xiao J. (2010). *In vivo* structure of the *E. coli* FtsZ-ring revealed by photoactivated localization microscopy (PALM). PLoS One.

[148] Hensel Z., Weng X., Lagda A.C., Xiao J. (2013). Transcription-factor-mediated DNA looping probed by high-resolution, single-molecule imaging in live *E. coli* cells. PLoS Biol..

[149] Lee S.F., Thompson M.A., Schwartz M.A., Shapiro L., Moerner W.E. (2011). Super-resolution imaging of the nucleoid-associated protein HU in *Caulobacter crescentus*. Biophys. J..

[150] Hammar P., Leroy P., Mahmutovic A., Marklund E.G., Berg O.G., Elf J. (2012). The *lac* repressor displays facilitated diffusion in living cells. Science.

